# HyperModules: identifying clinically and phenotypically significant network modules with disease mutations for biomarker discovery

**DOI:** 10.1093/bioinformatics/btu172

**Published:** 2014-04-08

**Authors:** Alvin Leung, Gary D. Bader, Jüri Reimand

**Affiliations:** The Donnelly Centre, University of Toronto, 160 College Street, M5S 3E1 Toronto, Ontario, Canada

## Abstract

**Summary:** Correlating disease mutations with clinical and phenotypic information such as drug response or patient survival is an important goal of personalized cancer genomics and a first step in biomarker discovery. HyperModules is a network search algorithm that finds frequently mutated gene modules with significant clinical or phenotypic signatures from biomolecular interaction networks.

**Availability and implementation:** HyperModules is available in Cytoscape App Store and as a command line tool at www.baderlab.org/Sofware/HyperModules.

**Contact:** Juri.Reimand@utoronto.ca or Gary.Bader@utoronto.ca

**Supplementary information:** Supplementary data are available at *Bioinformatics* online

## 1 INTRODUCTION

Establishing functional links between genetic variation and human disease is a key goal of cancer genome sequencing (Gonzalez-Perez *et al.*, 2013) and genome-wide association studies (Hardy and Singleton, 2009). Complex diseases like cancer are often driven by infrequent changes in multiple genes in pathways (Vogelstein *et al.*, 2013). Network analysis helps interpret mutations in systems context and find disease genes, pathways and biomarkers for precision medicine (Barabasi *et al.*, 2011).

Discovery of modules (subnetworks) in biological networks helps isolate systems with disease-related properties and reduces interactome complexity. A growing number of methods are available for this purpose. A landmark paper combines gene expression signatures with protein–protein interactions (PPI) to find predictive modules of cancer outcome (Chuang *et al.*, 2007). The NETBAG method studies genetic associations and copy number variants to find autism-related modules (Gilman *et al.*, 2011). HotNet detects frequently mutated pathways in networks (Vandin *et al.*, 2011, 2012). Net-Cox builds prognostic cancer signatures in network analysis of gene expression data (Zhang *et al.*, 2013). The Reactome FI Cytoscape plugin uncovers prognostic gene modules from networks and gene expression data (Wu and Stein, 2012). Network-based stratification predicts tumor subtypes from mutations in network regions (Hofree *et al.*, 2013). Such modules maximize a feature of genes such as differential expression, disease mutation frequency or enrichment of interactions.

Because clinical profiles of patients are increasingly available in cancer genomics efforts such as the The Cancer Genome Atlas (TCGA) pan-cancer project (Weinstein *et al.*, 2013), new methods are needed to discover multivariate biomarkers in networks. We recently analyzed cancer mutations in phosphorylation signaling and found that kinase–substrate networks are informative of patient survival and therapy response (Reimand and Bader, 2013; Reimand *et al.*, 2013). In particular, we found network modules with rare mutations in ovarian cancer patients with improved prognosis. We created the HyperModules method to systematically discover clinically correlated modules from gene and protein networks (Reimand and Bader, 2013) based on our earlier work on functional subnetwork discovery (Altmae *et al.*, 2012; Reimand *et al.*, 2008). Here we present the previously unavailable software in open-source Java as a command line tool for automated work and a Cytoscape app for interactive graphical analysis.

## 2 SOFTWARE

HyperModules assumes that clinically informative mutations of complex disease occur in systems of closely interacting genes. The greedy network search algorithm focuses on a local network area, defined by a central seed node (a mutated gene) and its surrounding subnetwork. All mutated genes are sequentially considered as seeds in module discovery. Search starts from the seed and grows the module toward increased benefit by adding connected genes that best improve clinical significance. This objective is driven by statistical tests where patients defined by the module are compared with other patients. Categorical clinical variables are studied with Fisher’s exact test and survival times with log-rank test. Cox regression is currently not supported; however, we plan to add this feature in the future. To establish statistical significance of detected modules, we build a null distribution by searching networks with permuted gene names. Each module of the true network is quantified with an empirical *P*-value reflecting the fraction of seed-specific modules from shuffled networks exceeding the significance of the true module. This removes artifacts of the greedy strategy and corrects for topological features such as highly connected nodes.

**Fig. 1.**
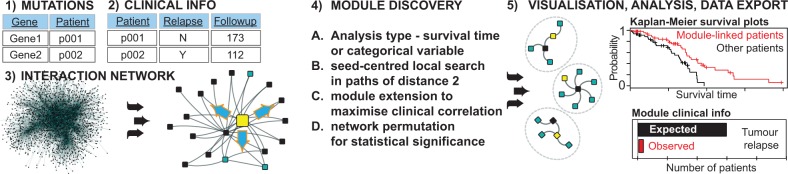
HyperModules requires three inputs—(**1**) mutated genes in patients, (**2**) patient clinical information and (**3**) protein or gene network. Search is performed for all mutated genes as seeds (**4**). Network visualization, clinical variable statistics and data export facilitate further analysis (**5**)

The analysis pipeline is outlined in Figure 1. Interaction networks are loaded into Cytoscape using standard features (Shannon *et al.*, 2003). HyperModules requires gene mutations and patient clinical information in two tables. The user selects type of clinical analysis and columns in data table (survival time or variable such as tumor relapse). Survival analysis requires follow-up time and vital status of patients. Detected modules are studied further with network visualization, survival curves and data export. We tested HyperModules on protein networks to find survival modules with cancer mutations. We extracted three human PPI networks of variable size from iRefWeb (Turner *et al.*, 2010), and five cancer mutation datasets from the International Cancer Genome Consortium portal version 12 (Supplementary Fig. S1). For example, network analysis of 30 000 interactions with 121 liver cancer patients, 686 mutated genes and 10 000 permutations takes 10 min on an 8-core computer with 16 GB RAM. HyperModules is thus applicable to a range of networks and mutation datasets.

## 3 EXAMPLE ANALYSIS

An example dataset is provided in Supplementary File S1. It comprises 183 ovarian cancer patients from the TCGA study (Cancer Genome Atlas Research Network, 2008) and the network of 4823 kinase–substrate interactions from our earlier study (Reimand and Bader, 2013). The ovarian cancer mutations are restricted to 163 proteins with single nucleotide variants affecting protein phosphorylation sites or kinase domains. Two sets of modules were computed with 10 000 network permutations and are shown in Supplementary Figures S2–S3. First, the search for survival correlations in the kinase–substrate network with log-rank test identified 19 modules, where associated patients have significantly different survival rates compared with other patients in the cohort (empirical *P* ≤ 0.05). Second, the categorical variable search with Fisher’s exact test revealed fivemodules with significant enrichment of alive patients (empirical *P* ≤ 0.05). The modules are also summarized in Supplementary Table S1.

## 4 DISCUSSION

HyperModules is a biological network-mining algorithm that reveals modules of interacting genes with clinically informative disease mutations. Diverse biomolecular interaction networks can be analyzed, including PPI networks, gene regulatory networks and curated biological pathways. Disease mutations are also broadly defined. Although we initially studied cancer point mutations, other types of alterations such as copy number and gene expression changes can be used. HyperModules finds correlations with groups of genes where mutations may be infrequent but the signature strengthens through network integration. Such modules are not often directly usable as biomarkers because of small sample size; however, we believe that our approach helps discover genes and pathways as potential multivariate biomarkers for further experiments.

## Supplementary Material

Supplementary Data
