# Supplementary material for: HyperModules: identifying clinically and phenotypically significant network modules with disease mutations for biomarker discovery
Source: Bioinformatics. 2014 Apr 8;30(15):2230–2. doi: 10.1093/bioinformatics/btu172 (PMC4103591; doi:10.1093/bioinformatics/btu172)
Supplement: Supplementary Data [file supp_30_15_2230__index.html]

HyperModules: identifying clinically and phenotypically significant network modules with disease mutations for biomarker discovery — Supplementary Data 

# HyperModules: identifying clinically and phenotypically significant network modules with disease mutations for biomarker discovery

## Supplementary Data

files

**Files in this Data Supplement:**

- Supplementary Data - pdf file
- Supplementary Data - zip file
