# Supplementary material for: HyperModules: identifying clinically and phenotypically significant network modules with disease mutations for biomarker discovery
Source: Bioinformatics. 2014 Apr 8;30(15):2230–2. doi: 10.1093/bioinformatics/btu172 (PMC4103591; doi:10.1093/bioinformatics/btu172)
Supplement: Supplementary Data [file supp_btu172_Supplementary_Information_-_tables_and_figures.pdf]

SUPPLEMENTARY INFORMATION:
HyperModules: identifying clinically and phenotypically significant network modules with disease mutations for biomarker discovery

ALVIN LEUNG[1], GARY D. BADER[1,*], JÜRI REIMAND[1,*]

1 - The Donnelly Centre, University of Toronto
* - Juri.Reimand@utoronto.ca, Gary.Bader@utoronto.ca

HYPERMODULES RUNTIME

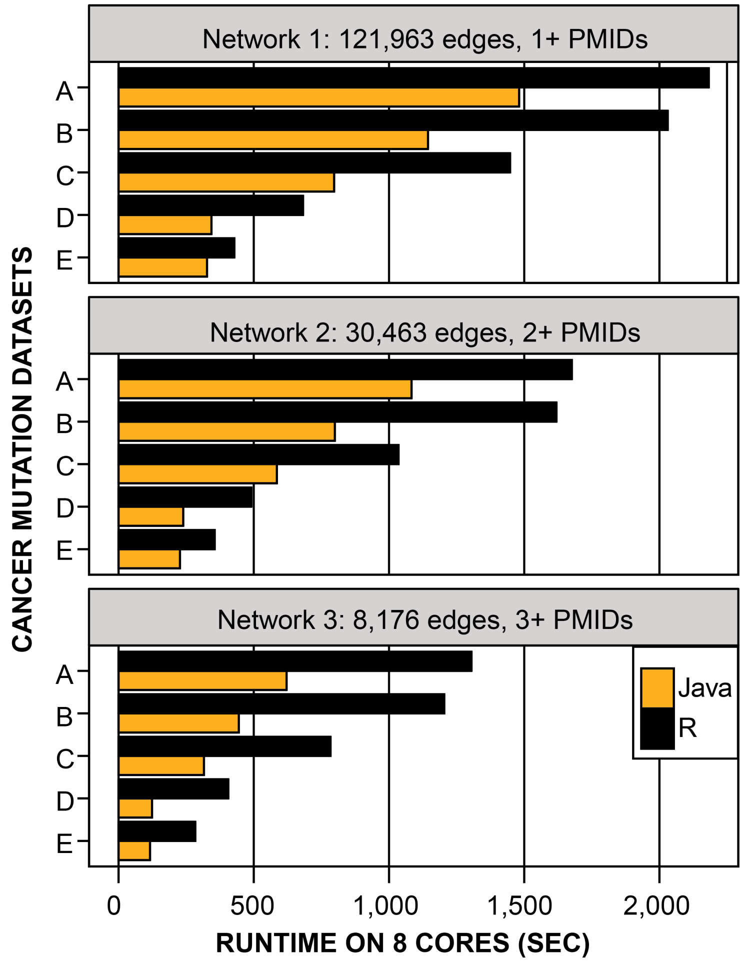

<u>Cancer mutations (seed genes, patients)</u>
A: glioblastoma (s=1231, p=93)
B: leukemia (s=939, p=241)
C: Liver cancer (s=686, p=121)
D: Ovarian cancer (s=292, p=568)
E: Pancreatic cancer (s=278, p=104)

Supplementary Figure 1. HyperModules runtime is shown for different protein-protein interaction (PPI) networks from iRefWeb and cancer mutation datasets from the ICGC cancer genomics portal. PPI networks were filtered based on confidence of interaction, with number of publications (PMIDs) that support the interaction. Comparison is shown between the original HyperModules R code and the newly developed Java code in the Cytoscape app and the command line software. A computer with Intel Core i7 3.5GHz CPU and 16 GB of RAM was used for runtime estimates.

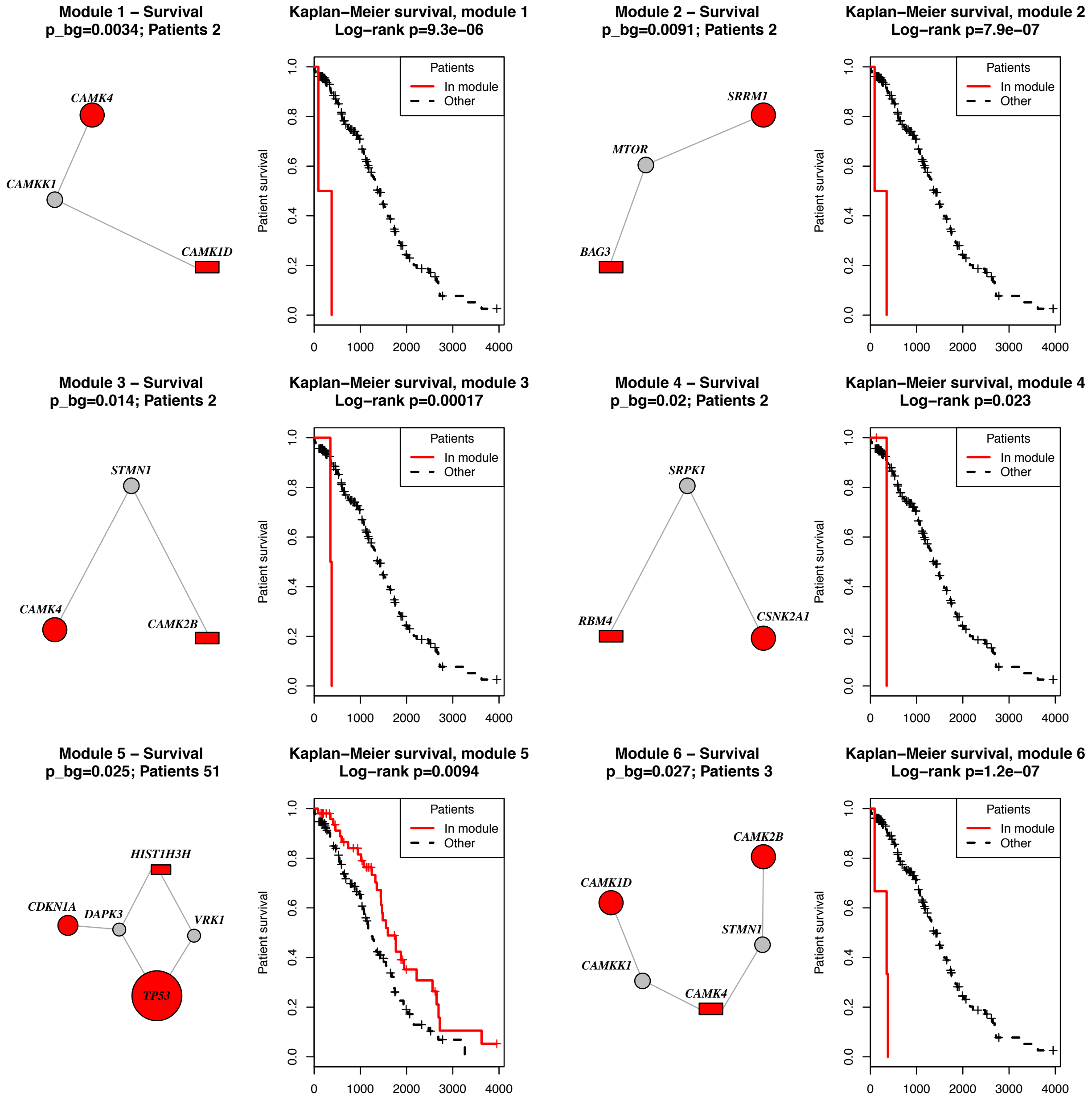

Supplementary Figure 2. HyperModules example of modules with significant survival correlation. 19 interaction survival-correlated modules with patients of the ovarian cancer dataset result from analysis of phosphorylation-associated mutations in the kinase-substrate network (modules 1-6 are shown). Log-rank test is used to search for modules where module-associated patients have significantly different survival profiles compared to other patients who carry no mutations in the module of interest. Network visualisations of modules and Kaplan-Meier survival curves are sorted left-right, top-bottom according to statistical significance. Background p-value (p_bg) is estimated from 10,000 network permutations. Genes in the module are color-coded (red – mutated gene; gray – non-mutated gene) and rectangles indicate seed genes. Log-rank p-value is shown above Kaplan-Meier plots.

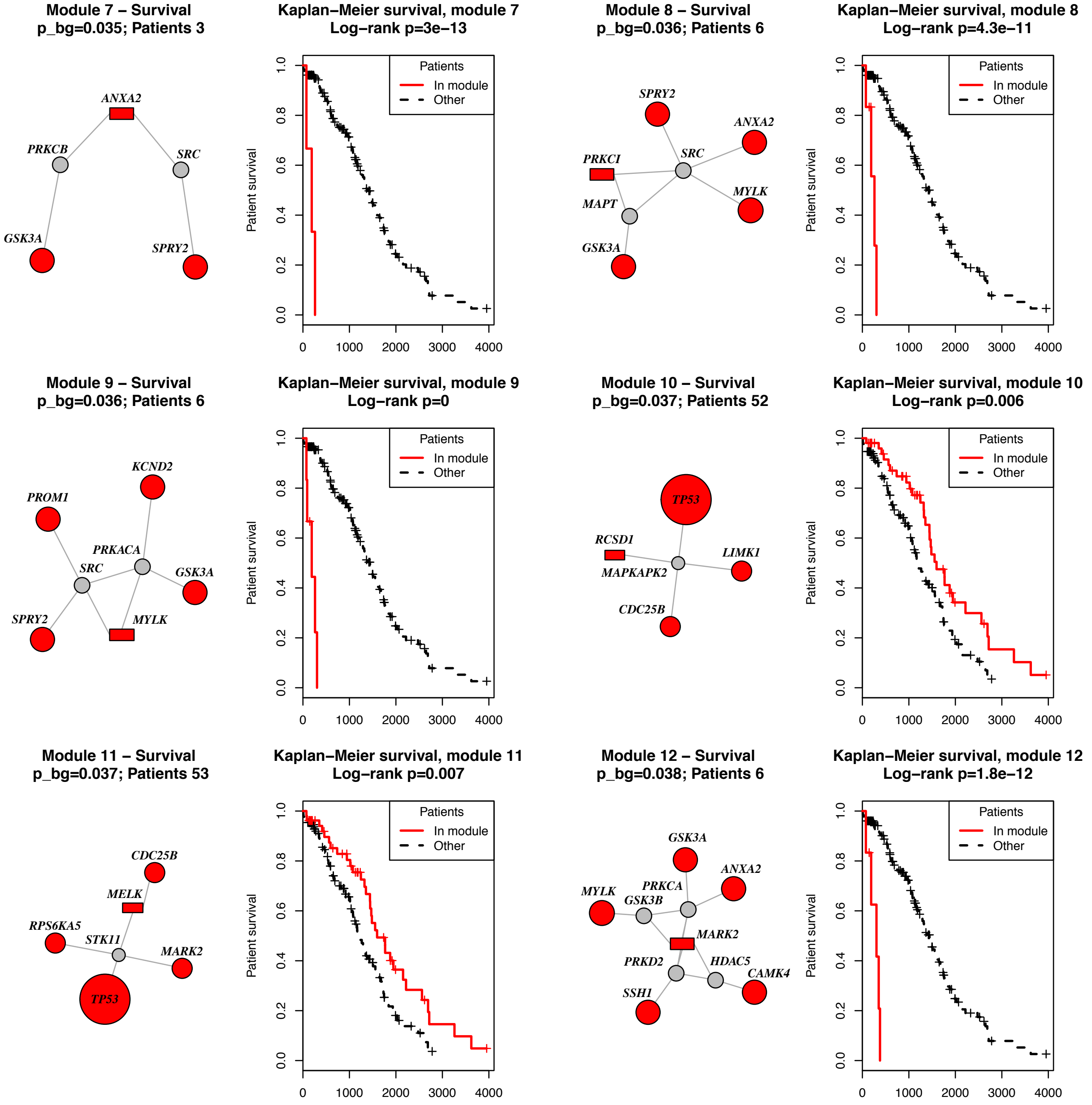

Supplementary Figure 2. Continued, modules 7-12 are shown.

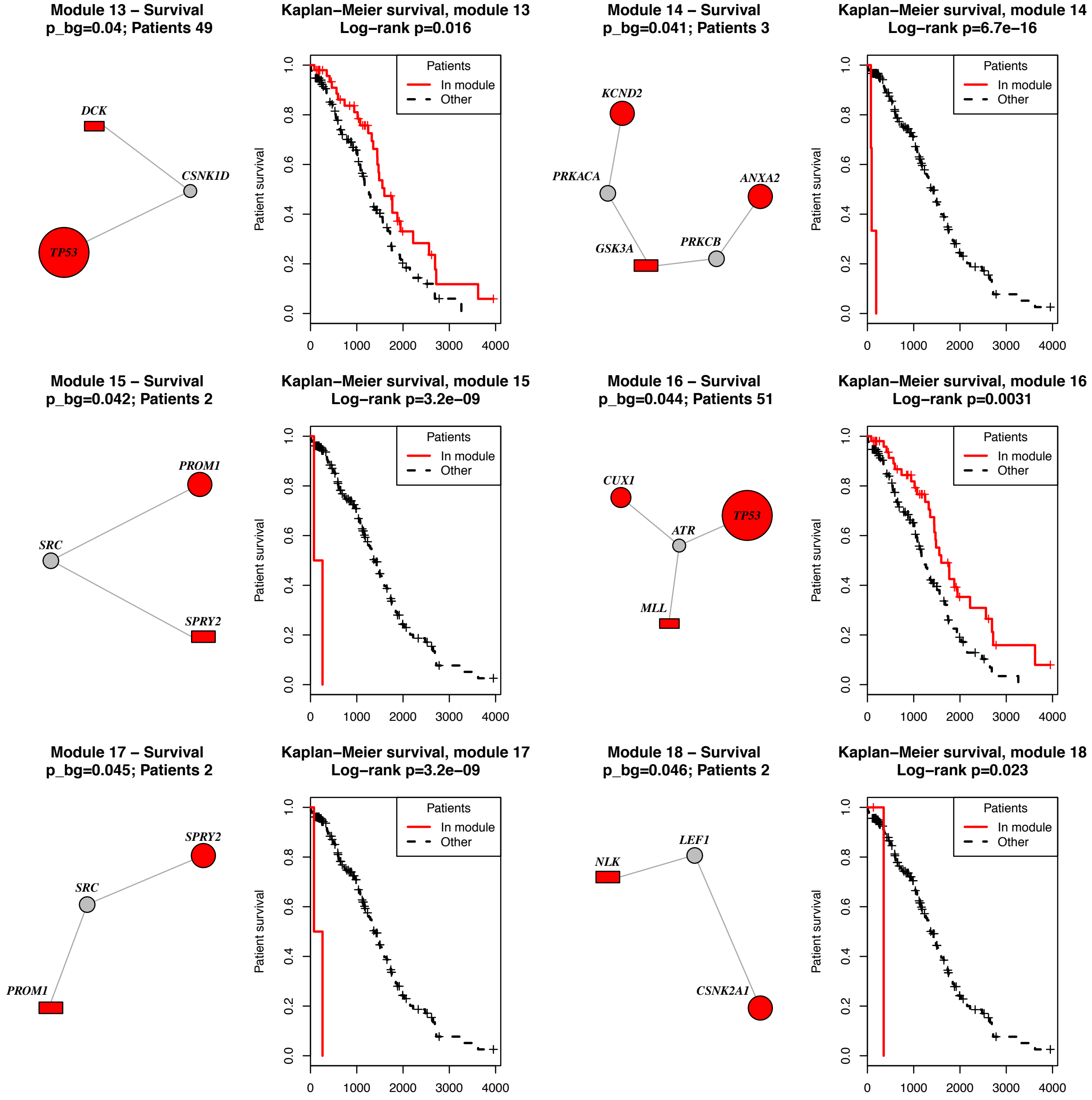

Supplementary Figure 2. Continued, modules 13-18 are shown.

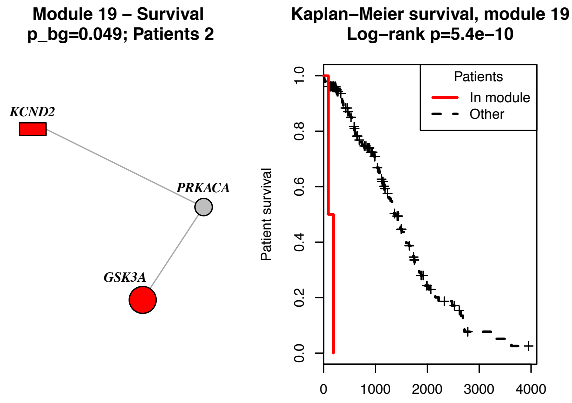

Supplementary Figure 2. Continued, module 19 is shown.

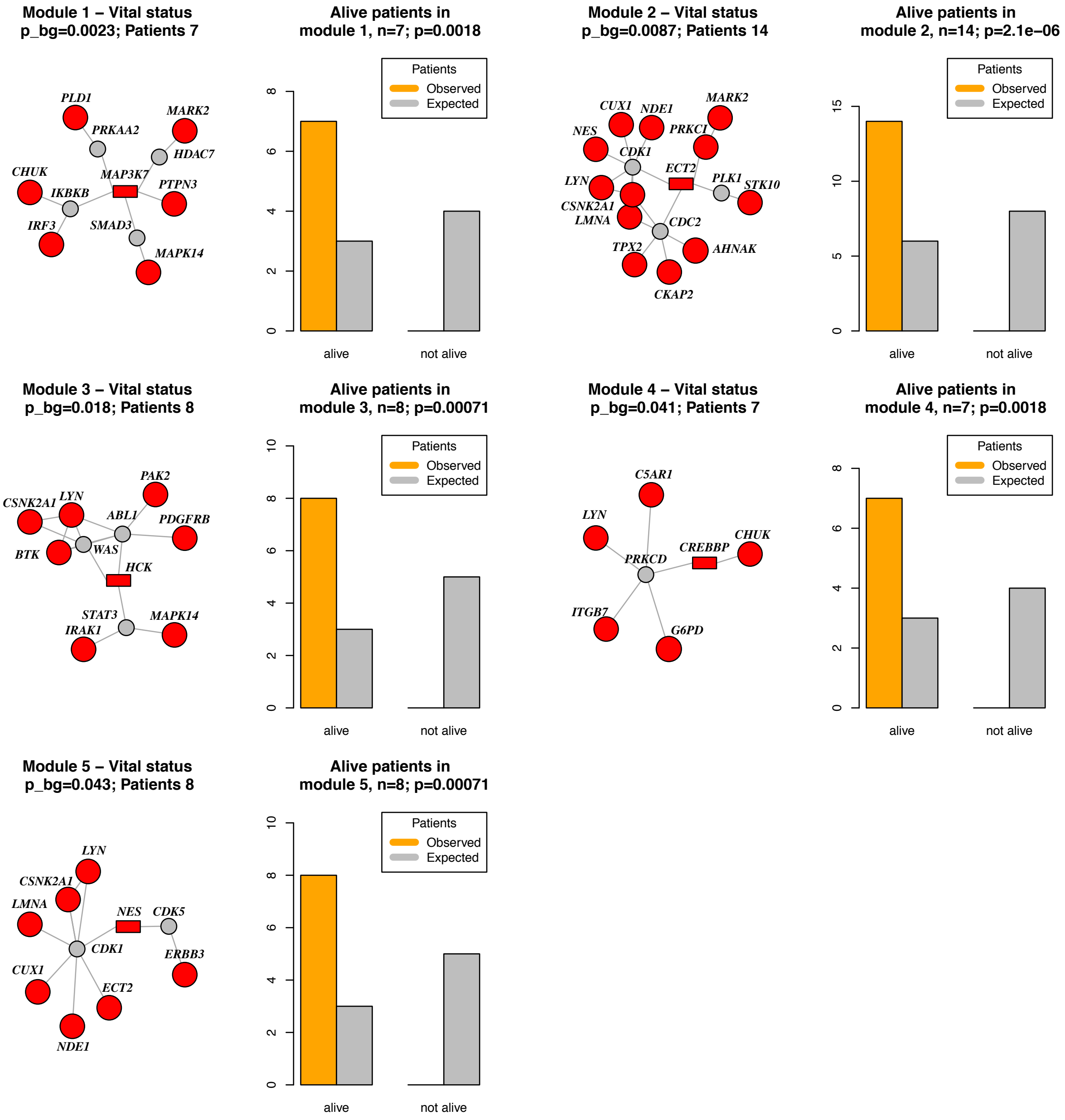

Supplementary Figure 3. HyperModules example of modules with enriched clinical features. Five interaction modules with enriched alive patients of the ovarian cancer dataset result from analysis of phosphorylation-associated mutations in the kinase-substrate network. Fisher's exact test is used to search for modules where module-associated patients are more frequently alive compared to other patients who carry no mutations in the module of interest. Network visualisations of modules and observed/expected barplots are sorted left-right, top-bottom according to statistical significance. Background p-value (p_bg) is estimated from 10,000 network permutations. Genes in the module are color-coded (red – mutated gene; gray – non-mutated gene) and rectangles indicate seed genes. Expected numbers of patients are sampled robustly from the binomial distribution, Fisher's exact p-value is shown above barplots.

Supplementary Table 1. Example modules from HyperModules analysis.

Analysis	Seed	Module genes	*P*_test	*P*_bg	# patients	Module score	Patient list
Survival	CAMK1D	CAMK1D CAMK4 CAMKK1	9.33E-06	0.0034	2	-0.94593	patient46 patient7
Survival	BAG3	BAG3 MTOR SRRM1	7.91E-07	0.0091	2	-1.0227	patient42 patient7
Survival	CAMK2B	CAMK2B CAMK4 STMN1	1.71E-04	0.0136	2	-0.94593	patient42 patient46
Survival	RBM4	CSNK2A1 RBM4 SRPK1	0.02269	0.0204	2	-1.0227	patient10 patient42
Survival	HIST1H3H	CDKN1A DAPK3 HIST1H3H TP53 VRK1	0.00942	0.0254	51	0.34527	patient10 patient100 patient103 patient109 patient113 patient115 patient118 patient119 patient123 patient126 patient131 patient132 patient134 patient135 patient139 patient142 patient149 patient154 patient155 patient157 patient158 patient159 patient16 patient161 patient164 patient169 patient173 patient174 patient175 patient177 patient178 patient181 patient182 patient21 patient24 patient25 patient36 patient39 patient42 patient47 patient49 patient50 patient6 patient60 patient63 patient69 patient76 patient81 patient90 patient91 patient97
Survival	CAMK4	CAMK1D CAMK2B CAMK4 CAMKK1 STMN1	1.21E-07	0.027	3	-1.0278	patient42 patient46 patient7
Survival	ANXA2	ANXA2 GSK3A PRKCB SPRY2 SRC	2.96E-13	0.0354	3	-1.6468	patient22 patient35 patient5
Survival	PRKCI	ANXA2 GSK3A MAPT MYLK PRKCI SPRY2 SRC	4.34E-11	0.0356	6	-1.6549	patient14 patient20 patient22 patient35 patient38 patient5
Survival	MYLK	GSK3A KCND2 MYLK PRKACA PROM1 SPRY2 SRC	0	0.0356	6	-1.6549	patient14 patient22 patient35 patient38 patient5 patient7
Survival	RCSD1	CDC25B LIMK1 MAPKAPK2 RCSD1 TP53	0.00599	0.037	52	0.41919	patient10 patient100 patient103 patient109 patient113 patient115 patient118 patient119 patient122 patient123 patient126 patient131 patient132 patient134 patient135 patient139 patient142 patient149 patient154 patient155 patient157 patient158 patient159 patient16 patient161 patient164 patient169 patient173 patient174 patient177 patient178 patient180 patient181 patient182 patient21 patient24 patient25 patient36 patient42 patient47 patient49 patient50 patient6 patient60 patient63 patient69 patient76 patient81 patient82 patient90 patient91 patient97

Analysis	Seed	Module genes	*P*_test	*P*_bg	# patients	Module score	Patient list
Survival	MELK	CDC25B MARK2 MELK RPS6KA5 STK11 TP53	0.00704	0.0372	53	0.34527	patient10 patient100 patient103 patient109 patient113 patient115 patient118 patient119 patient123 patient126 patient131 patient132 patient134 patient135 patient139 patient14 patient142 patient149 patient154 patient155 patient157 patient158 patient159 patient16 patient161 patient164 patient168 patient169 patient173 patient174 patient177 patient178 patient180 patient181 patient182 patient21 patient24 patient25 patient36 patient42 patient47 patient49 patient5 patient50 patient6 patient60 patient63 patient69 patient76 patient81 patient90 patient91 patient97
Survival	MARK2	ANXA2 CAMK4 GSK3A GSK3B HDAC5 MARK2 MYLK PRKCA PRKD2 SSH1	1.80E-12	0.0384	6	-1.183	patient14 patient22 patient38 patient42 patient46 patient5
Survival	DCK	CSNK1D DCK TP53	0.01647	0.0396	49	0.34527	patient10 patient100 patient103 patient109 patient113 patient115 patient118 patient119 patient123 patient126 patient131 patient132 patient134 patient135 patient139 patient142 patient149 patient154 patient155 patient157 patient158 patient159 patient16 patient161 patient164 patient169 patient173 patient174 patient177 patient178 patient181 patient182 patient21 patient24 patient25 patient36 patient42 patient47 patient49 patient50 patient6 patient60 patient63 patient69 patient76 patient81 patient90 patient91 patient97
Survival	GSK3A	ANXA2 GSK3A KCND2 PRKACA PRKCB	6.66E-16	0.041	3	-2.3777	patient22 patient5 patient7
Survival	SPRY2	PROM1 SPRY2 SRC	3.23E-09	0.0418	2	-1.3228	patient35 patient5
Survival	MLL	ATR CUX1 MLL TP53	0.00309	0.0435	51	0.34986	patient10 patient100 patient103 patient109 patient113 patient115 patient118 patient119 patient123 patient126 patient131 patient132 patient134 patient135 patient139 patient142 patient149 patient154 patient155 patient157 patient158 patient159 patient16 patient161 patient164 patient169 patient173 patient174 patient177 patient178 patient179 patient181 patient182 patient21 patient24 patient25 patient36 patient42 patient47 patient49 patient50 patient6 patient60 patient63 patient69 patient76 patient81 patient84 patient90 patient91 patient97
Survival	PROM1	PROM1 SPRY2 SRC	3.23E-09	0.0454	2	-1.3228	patient35 patient5
Survival	NLK	CSNK2A1 LEF1 NLK	0.02269	0.0456	2	-1.0227	patient10 patient42
Survival	KCND2	GSK3A KCND2 PRKACA	5.39E-10	0.049	2	-1.6417	patient22 patient7
Vital	MAP3K7	CHUK HDAC7 IKBKB IRF3 MAP3K7 MAPK14 MARK2 PLD1 PRKAA2 PTPN3 SMAD3	0.0018	0.0023	7	1000 (Inf)	patient110 patient113 patient127 patient129 patient14 patient158 patient93

Analysis	Seed	Module genes	*P*_test	*P*_bg	# patients	Module score	Patient list
Vital	ECT2	AHNAK CDC2 CDK1 CKAP2 CSNK2A1 CUX1 ECT2 LMNA LYN MARK2 NDE1 NES PLK1 PRKCI STK10 TPX2	2.11E-06	0.0087	14	1000 (Inf)	patient10 patient113 patient129 patient14 patient159 patient166 patient20 patient21 patient32 patient39 patient48 patient52 patient56 patient84
Vital	HCK	ABL1 BTK CSNK2A1 HCK IRAK1 LYN MAPK14 PAK2 PDGFRB STAT3 WAS	7.06E-04	0.0185	8	1000 (Inf)	patient10 patient136 patient15 patient158 patient166 patient172 patient179 patient37
Vital	CREBBP	C5AR1 CHUK CREBBP G6PD ITGB7 LYN PRKCD	0.0018	0.0413	7	1000 (Inf)	patient107 patient110 patient118 patient127 patient129 patient166 patient37
Vital	NES	CDK1 CDK5 CSNK2A1 CUX1 ECT2 ERBB3 LMNA LYN NDE1 NES	7.06E-04	0.0432	8	1000 (Inf)	patient10 patient129 patient144 patient166 patient32 patient39 patient52 patient84
